# 2-Amino-4-nitro­phenol–1-(2,4,6-trihy­droxy­phen­yl)ethanone (1/1)

**DOI:** 10.1107/S1600536812017497

**Published:** 2012-04-25

**Authors:** Can Kocabıyık, Hümeyra Paşaoğlu, Taşkın Basılı, Erbil Ağar

**Affiliations:** a Ondokuz Mayıs University, Faculty of Arts and Sciences, Department of Physics, 55139 Kurupelit Samsun, Turkey; b Ondokuz Mayıs University, Faculty of Arts and Sciences, Department of Chemistry, 55139 Kurupelit Samsun, Turkey

## Abstract

In the title compound, C_6_H_6_N_2_O_3_·C_8_H_8_O_4_, the 2-amino-4-nitro­phenol (ANP) and 1-(2,4,6-trihy­droxy­phen­yl)ethanone (THA) mol­ecules are both nearly planar, with r.m.s. deviations of 0.0630 and 0.0313 Å, respectively. The angle between the least-squares planes of THA and ANP is 48.99 (2)°. In THA, an intra­molecular O—H⋯O hydrogen bond generates an *S*(6) ring motif. In the crystal, N—H⋯O, O—H⋯O and O—H⋯N hydrogen bonds lead to the formation of a three-dimensional network. There are also inter­molecular π–π inter­actions between the benzene rings of ANP–ANP and of THA–THA mol­ecules, with centroid–centroid distances of 3.5313 (14) and 3.8440 (16) Å, respectively. Weak C—O⋯π and N—O⋯π inter­actions also occur.

## Related literature

For the use of nitro­aromatics as inter­mediates in explosives, dyestuffs, pesticides and organic synthesis, see: Yan *et al.* (2006 ▶). For the occurrence of nitro­aromatics in industrial wastes and as direct pollutants in the environment, see: Yan *et al.* (2006 ▶); Soojhawon *et al.* (2005 ▶). For graph-set motifs, see: Bernstein *et al.* (1995 ▶). For related structures, see: Tanak *et al.* (2009 ▶, 2010 ▶); Ali *et al.* (2008 ▶); Bi *et al.* (2009 ▶); Garden *et al.* (2004 ▶); Serdiuk *et al.* (2011 ▶).
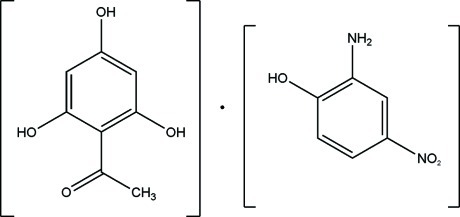


## Experimental

### 

#### Crystal data

C_6_H_6_N_2_O_3_·C_8_H_8_O_4_
*M* *_r_* = 322.27
Monoclinic, 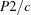

*a* = 7.7255 (6) Å
*b* = 13.2184 (11) Å
*c* = 15.8335 (12) Å
β = 118.148 (5)°
*V* = 1425.67 (19) Å^3^
*Z* = 4
Mo *K*α radiation
μ = 0.12 mm^−1^
*T* = 296 K
0.80 × 0.35 × 0.09 mm

#### Data collection

Stoe IPDS 2 diffractometer
Absorption correction: integration (*X-RED*; Stoe & Cie, 2002 ▶) *T*_min_ = 0.942, *T*_max_ = 0.992
15333 measured reflections
2961 independent reflections
1841 reflections with *I* > 2σ(*I*)
*R*_int_ = 0.065

#### Refinement

*R*[*F* ^2^ > 2σ(*F* ^2^)] = 0.046
*wR*(*F* ^2^) = 0.098
*S* = 0.97
2961 reflections
252 parameters
H atoms treated by a mixture of independent and constrained refinement
Δρ_max_ = 0.15 e Å^−3^
Δρ_min_ = −0.24 e Å^−3^

### 

Data collection: *X-AREA* (Stoe & Cie, 2002 ▶); cell refinement: *X-AREA*; data reduction: *X-RED32* (Stoe & Cie, 2002 ▶); program(s) used to solve structure: *SHELXS97* (Sheldrick, 2008 ▶); program(s) used to refine structure: *SHELXL97* (Sheldrick, 2008 ▶); molecular graphics: *ORTEP-3 for Windows* (Farrugia, 1997 ▶) and *Mercury* (Macrae *et al.*, 2006 ▶); software used to prepare material for publication: *WinGX* (Farrugia, 1999 ▶).

## Supplementary Material

Crystal structure: contains datablock(s) I, global. DOI: 10.1107/S1600536812017497/kj2199sup1.cif

Structure factors: contains datablock(s) I. DOI: 10.1107/S1600536812017497/kj2199Isup2.hkl

Supplementary material file. DOI: 10.1107/S1600536812017497/kj2199Isup3.cml

Additional supplementary materials:  crystallographic information; 3D view; checkCIF report

## Figures and Tables

**Table 1:** Hydrogen-bond geometry (Å, °) *Cg*1 and *Cg*2 are the centroids of the C7–C12 and C1–C6 rings, respectively.

*D*—H⋯*A*	*D*—H	H⋯*A*	*D*⋯*A*	*D*—H⋯*A*
N2—H2*A*⋯O6^i^	0.93 (3)	2.28 (3)	3.067 (2)	141.5 (19)
N2—H2*B*⋯O6^ii^	0.93 (3)	2.36 (3)	3.241 (3)	157 (2)
O3—H3*A*⋯N1^iii^	0.84 (3)	2.59 (3)	3.358 (3)	153 (3)
O3—H3*A*⋯O1^iii^	0.84 (3)	2.39 (3)	2.953 (3)	125 (3)
O3—H3*A*⋯O2^iii^	0.84 (3)	2.13 (3)	2.975 (3)	178 (3)
O4—H4⋯N2	0.86 (3)	1.94 (3)	2.784 (2)	166 (2)
O5—H5*A*⋯O7^i^	0.88 (3)	1.87 (3)	2.748 (2)	175 (3)
O6—H6⋯O7	0.92 (3)	1.64 (3)	2.478 (2)	150 (2)
N1—O2⋯*Cg*2^iv^	1.22 (1)	3.82 (1)	3.599 (3)	70 (1)
C13—O7⋯*Cg*1^v^	1.25 (1)	3.52 (1)	3.722 (3)	89 (1)

Symmetry codes: (i) 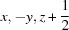; (ii) 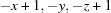; (iii) 
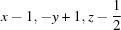
; (iv) 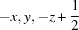; (v) 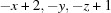.

**Fig. 1.**
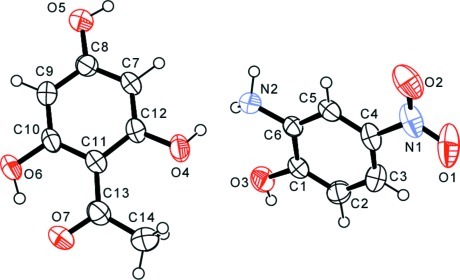
The molecular structure of the title compound, showing the atom-numbering scheme and 50% probability displacement ellipsoids.

**Fig. 2.**
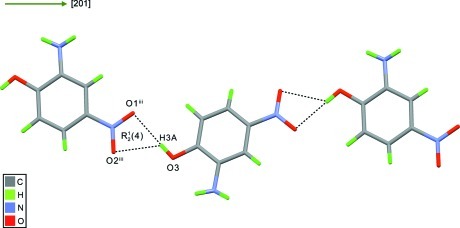
H-bonds (dotted lines) form R12(4) rings and C(8) chains running in the [201] direction.

**Fig. 3.**
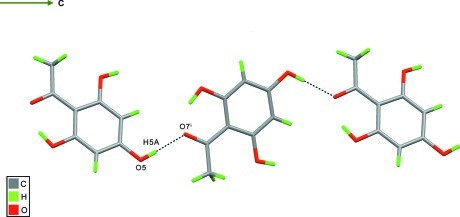
The C(8) chain structure running along the [001] direction formed by H-bonds (dotted lines) between 2,4,6-trihydroxyacetephenone molecules.

**Fig. 4.**
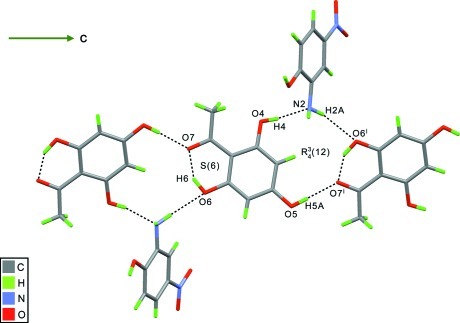
H-bonds (dotted lines) form R34(12) and S(6)-rings.

## supplementary crystallographic information

### Comment

Nitroaromatics are widely used either as materials or as intermediates in explosives, dyestuffs, pesticides and organic synthesis (Yan *et al.*, 2006). Nitroaromatics occur as industrial wastes and direct pollutants in the environment, and are relatively soluble in water and detectable in rivers, ponds and soil (Yan *et al.*, 2006; Soojhawon *et al.*, 2005).

The title compound (Fig. 1) crystallizes in the monoclinic space group P2/c with *Z* = 4 in the unit cell. The asymmetric unit in the crystal structure therefore contains only one formula unit. The bond lengths and angles of the ANP and THA moieties have normal values. The C4—N1 [1.448 (3) Å] and C13—C14 [1.486 (3) Å] bond distances are comparable to those observed in related structures (Ali *et al.*, 2008; Tanak *et al.*, 2009; Tanak *et al.*, 2010; Bi *et al.*, 2009; Garden *et al.*, 2004; Serdiuk *et al.* 2011). The ANP and THA molecules are almost planar with the maximum deviations, -0.0694 (18) Å for atom O1 in the ANP and -0.152 (2) Å for atom C14 in the THA molecules. The dihedral angle between these rings is 48.99 (2)°.

The crystal packing of the title compound is stabilized by non-covalent hydrogen bond, π-π and X—Y···π-ring interactions. It can be seen from Fig. 2 that neighbouring ANP moieties are linked by O3—H3A···O1^iii^ and O3—H3A···O2^iii^ (iii: *x* - 1, -*y* + 1, *z* - 1/2) hydrogen bonds to form C(8) chains in direction [201], producing *R*^1^_2_(4) rings (Bernstein *et al.*, 1995). In addition, THA moieties are mutually connected to each other by O5—H5A···O7^i^ hydrogen bonds (i: *x*, -*y*, *z* + 1/2), forming a C(8) chain running in direction [001] (Fig. 3). These two chains are further connected by N2—H2A···O6^i^, N2—H2B···O6^ii^ (ii: -*x* + 1, -*y*, -*z* + 1) and O4—H4···N2 hydrogen bonds between ANP and THA moieties. The arrangement of ANP and THA gives rise to *R*^2^_2_(8) and *R*^3^_4_(12) rings. The N2 (in ANP) and O6 (in THA) atoms act as both donor and acceptor. Finally, the intra-molecular O6—H6···O7 hydrogen bond of THA generates an S(6) ring motif (Fig. 4).

In the extended structure of the compound, there are weak π-π and X—Y···π-ring interactions. The intermolecular π-π contact occurs between the two symmetry-related ANP (ring A) rings of neighboring molecules. Ring A is oriented in such a way that the distance between the ring centroids is 3.8440 (16) Å. The other π-π interaction is between THA (ring B) rings, with a distance of 3.5313 (14) Å between the ring centroids. Rings A and B are also involved in intermolecular N—O···π and C—O···π interactions through N atom of ANP and C atom of THA. With regard to the N—O···π contact, for two neighboring B rings,the distance between atom O2 and the center of ring B (CgB) is 3.822 (2) Å and the N1—O2···CgB angle is 70.29 (15)°. In addition, there are also C—O···π interactions between C13—O7 and A rings, which can be characterized by the O7···CgA distance of 3.521 (3) Å and the C13—O7···CgA angle of 89.33 (15)°.

### Experimental

1-(2,4,6-trihydroxyphenyl)ethanone-2-amino-4-nitrophenol (1/1) was prepared by refluxing a mixture of a solution containing 2,4,6-trihydroxyacetephenone (18,6 mg, 0,1 mmol) in ethanol (25 ml) and a solution containing 2-amino-4-nitrophenol (15,4 mg, 0,1 mmol) in ethanol (20 ml). The reaction mixture was stirred for 4 h under reflux. Single crystals of the title compound for X-ray analysis were obtained by slow evaporation of an ethanol solution (Yield 74%; m.p 442.-446 K).

### Refinement

The H atoms of the methyl group were positioned geometrically and treated using a riding model, with *U*_iso_(H) = 1.5*U*_eq_(C). All other H atoms were located in a difference map and refined freely.

### Crystal data

**Table:**

C_6_H_6_N_2_O_3_·C_8_H_8_O_4_	*F*(000) = 672
*M**_r_* = 322.27	*D*_x_ = 1.501 Mg m^−^^3^
Monoclinic, *P*2/*c*	Mo *K*α radiation, λ = 0.71073 Å
Hall symbol: -P 2yc	Cell parameters from 12619 reflections
*a* = 7.7255 (6) Å	θ = 2.1–27.3°
*b* = 13.2184 (11) Å	µ = 0.12 mm^−^^1^
*c* = 15.8335 (12) Å	*T* = 296 K
β = 118.148 (5)°	Prism, yellow
*V* = 1425.67 (19) Å^3^	0.80 × 0.35 × 0.09 mm
*Z* = 4	

### Data collection

**Table:**

Stoe IPDS 2 diffractometer	2961 independent reflections
Radiation source: fine-focus sealed tube	1841 reflections with *I* > 2σ(*I*)
Graphite monochromator	*R*_int_ = 0.065
Detector resolution: 6.67 pixels mm^-1^	θ_max_ = 26.5°, θ_min_ = 2.1°
rotation method scans	*h* = −9→9
Absorption correction: integration (*X-RED*; Stoe & Cie, 2002)	*k* = −16→16
*T*_min_ = 0.942, *T*_max_ = 0.992	*l* = −19→19
15333 measured reflections	

### Refinement

**Table:**

Refinement on *F*^2^	Primary atom site location: structure-invariant direct methods
Least-squares matrix: full	Secondary atom site location: difference Fourier map
*R*[*F*^2^ > 2σ(*F*^2^)] = 0.046	Hydrogen site location: inferred from neighbouring sites
*wR*(*F*^2^) = 0.098	H atoms treated by a mixture of independent and constrained refinement
*S* = 0.97	*w* = 1/[σ^2^(*F*_o_^2^) + (0.0466*P*)^2^] where *P* = (*F*_o_^2^ + 2*F*_c_^2^)/3
2961 reflections	(Δ/σ)_max_ < 0.001
252 parameters	Δρ_max_ = 0.15 e Å^−^^3^
0 restraints	Δρ_min_ = −0.24 e Å^−^^3^

### Special details

**Table:**

Experimental. IR (KBr, cm^-1^): 3523 ν(OH)_THA_, 3383 ν(NH_2_)*_asym_*, 3353 ν(NH_2_)*_sym_*+ν(OH)_THA_, 3298 ν(OH)_THA_, 3090–3000 ν(CH), 2850–2700 ν(CH_3_), 1628 ν(C=O)+δ(OH)_THA_+ν(ring)_THA_, 1614 ν(NH_2_)+ν(ring)_ANP_, 1571 δ(NH_2_), 1524 ν(NO_2_)*_asym_*, 1496–1476 δ(CH)+δ(OH)_ANP_, 1364–1339 δ(CH_3_)+δ(OH)_THA_, 1311–1251 ν(NO_2_)+ν(CO)_ANP_, 1203–1167–1147 ν(ring)+δ(OH). UV/Visible (nm): 226 (2,212 Å; ε= 19230 *L* mol^-1^cm^-1^) and 288 (1,815 Å; ε= 15780 *L* mol^-1^cm^-1^) π→π^*^ transitions of benzene ring (E bands), 380 nm (0,345 Å; ε= 3000 *L* mol^-1^cm^-1^) π→π^*^ transition of aniline (E_2_ band).
Geometry. All e.s.d.'s (except the e.s.d. in the dihedral angle between two l.s. planes) are estimated using the full covariance matrix. The cell e.s.d.'s are taken into account individually in the estimation of e.s.d.'s in distances, angles and torsion angles; correlations between e.s.d.'s in cell parameters are only used when they are defined by crystal symmetry. An approximate (isotropic) treatment of cell e.s.d.'s is used for estimating e.s.d.'s involving l.s. planes.
Refinement. Refinement of *F*^2^ against ALL reflections. The weighted *R*-factor *wR* and goodness of fit *S* are based on *F*^2^, conventional *R*-factors *R* are based on *F*, with *F* set to zero for negative *F*^2^. The threshold expression of *F*^2^ > σ(*F*^2^) is used only for calculating *R*-factors(gt) *etc*. and is not relevant to the choice of reflections for refinement. *R*-factors based on *F*^2^ are statistically about twice as large as those based on *F*, and *R*- factors based on ALL data will be even larger.

### Fractional atomic coordinates and isotropic or equivalent isotropic displacement parameters (Å^2^)

**Table:**

	*x*	*y*	*z*	*U*_iso_*/*U*_eq_	
C1	0.5977 (3)	0.40750 (14)	0.66694 (13)	0.0394 (5)	
C2	0.6191 (4)	0.51128 (15)	0.67342 (16)	0.0498 (6)	
C3	0.7703 (4)	0.55477 (16)	0.75249 (17)	0.0520 (6)	
C4	0.8964 (3)	0.49206 (15)	0.82522 (14)	0.0456 (5)	
C5	0.8776 (3)	0.38806 (15)	0.82066 (14)	0.0411 (5)	
C6	0.7271 (3)	0.34480 (13)	0.74077 (13)	0.0361 (4)	
C7	0.7798 (3)	0.00373 (15)	0.64563 (14)	0.0411 (5)	
C8	0.7390 (3)	−0.09742 (14)	0.62254 (14)	0.0423 (5)	
C9	0.7120 (3)	−0.13396 (15)	0.53533 (15)	0.0461 (5)	
C10	0.7236 (3)	−0.06837 (14)	0.47110 (13)	0.0409 (5)	
C11	0.7631 (3)	0.03665 (14)	0.49051 (13)	0.0380 (5)	
C12	0.7933 (3)	0.06963 (14)	0.58187 (14)	0.0395 (5)	
C13	0.7601 (3)	0.10280 (15)	0.41713 (15)	0.0466 (5)	
C14	0.7792 (5)	0.21463 (17)	0.42704 (19)	0.0779 (9)	
H14A	0.7731	0.2432	0.3700	0.117*	
H14B	0.9027	0.2316	0.4810	0.117*	
H14C	0.6742	0.2414	0.4365	0.117*	
N1	1.0593 (3)	0.53726 (17)	0.90766 (15)	0.0622 (6)	
N2	0.7059 (3)	0.23928 (13)	0.72998 (14)	0.0473 (5)	
O1	1.0834 (3)	0.62880 (15)	0.91011 (15)	0.0944 (7)	
O2	1.1715 (3)	0.48199 (16)	0.97227 (13)	0.0774 (6)	
O3	0.4543 (3)	0.35925 (12)	0.59151 (11)	0.0571 (5)	
O4	0.8398 (3)	0.16790 (11)	0.60576 (12)	0.0587 (5)	
O5	0.7222 (3)	−0.16375 (12)	0.68295 (12)	0.0640 (5)	
O6	0.6904 (3)	−0.10661 (12)	0.38519 (11)	0.0579 (5)	
O7	0.7370 (3)	0.06669 (11)	0.33974 (10)	0.0605 (5)	
H2	0.532 (3)	0.5607 (15)	0.6211 (15)	0.053 (6)*	
H2A	0.764 (3)	0.2079 (17)	0.7899 (18)	0.061 (7)*	
H2B	0.575 (4)	0.2194 (19)	0.692 (2)	0.078 (9)*	
H3	0.790 (3)	0.6232 (17)	0.7574 (15)	0.050 (6)*	
H3A	0.375 (5)	0.404 (2)	0.556 (2)	0.100 (11)*	
H4	0.812 (4)	0.1827 (18)	0.6505 (19)	0.063 (8)*	
H5	0.963 (3)	0.3471 (14)	0.8687 (15)	0.042 (6)*	
H5A	0.734 (4)	−0.134 (2)	0.735 (2)	0.093 (10)*	
H6	0.702 (4)	−0.0539 (18)	0.3500 (18)	0.067 (8)*	
H7	0.802 (3)	0.0289 (14)	0.7084 (14)	0.041 (5)*	
H9	0.691 (3)	−0.2043 (18)	0.5206 (16)	0.060 (6)*	

### Atomic displacement parameters (Å^2^)

**Table:**

	*U*^11^	*U*^22^	*U*^33^	*U*^12^	*U*^13^	*U*^23^
C1	0.0367 (12)	0.0479 (11)	0.0311 (10)	−0.0011 (9)	0.0139 (9)	−0.0043 (8)
C2	0.0508 (15)	0.0448 (12)	0.0474 (13)	0.0077 (10)	0.0178 (12)	0.0048 (10)
C3	0.0570 (16)	0.0380 (12)	0.0619 (15)	0.0002 (10)	0.0287 (13)	−0.0089 (10)
C4	0.0387 (13)	0.0540 (12)	0.0407 (11)	−0.0053 (10)	0.0161 (10)	−0.0169 (9)
C5	0.0402 (13)	0.0514 (12)	0.0298 (10)	0.0053 (10)	0.0149 (10)	−0.0007 (9)
C6	0.0402 (12)	0.0384 (10)	0.0328 (10)	0.0000 (8)	0.0199 (9)	−0.0029 (8)
C7	0.0444 (13)	0.0483 (11)	0.0307 (10)	−0.0019 (9)	0.0178 (10)	−0.0075 (8)
C8	0.0452 (14)	0.0439 (11)	0.0382 (11)	0.0033 (9)	0.0199 (10)	0.0011 (8)
C9	0.0573 (15)	0.0368 (11)	0.0455 (12)	−0.0008 (9)	0.0254 (11)	−0.0066 (9)
C10	0.0435 (13)	0.0450 (11)	0.0325 (10)	0.0013 (9)	0.0165 (9)	−0.0100 (8)
C11	0.0346 (12)	0.0460 (11)	0.0335 (10)	−0.0017 (9)	0.0163 (9)	−0.0056 (8)
C12	0.0374 (13)	0.0435 (10)	0.0381 (11)	−0.0055 (9)	0.0183 (9)	−0.0108 (8)
C13	0.0477 (14)	0.0534 (12)	0.0426 (12)	−0.0058 (10)	0.0245 (11)	−0.0056 (9)
C14	0.126 (3)	0.0560 (14)	0.0640 (17)	−0.0166 (15)	0.0552 (18)	0.0019 (12)
N1	0.0463 (14)	0.0798 (15)	0.0543 (13)	−0.0063 (11)	0.0188 (11)	−0.0278 (11)
N2	0.0591 (14)	0.0416 (10)	0.0385 (10)	−0.0016 (9)	0.0208 (10)	0.0011 (8)
O1	0.0808 (15)	0.0721 (12)	0.1081 (17)	−0.0231 (10)	0.0262 (12)	−0.0512 (11)
O2	0.0540 (12)	0.1078 (14)	0.0477 (10)	−0.0066 (11)	0.0053 (9)	−0.0182 (10)
O3	0.0502 (11)	0.0598 (10)	0.0398 (9)	−0.0014 (8)	0.0035 (8)	−0.0051 (7)
O4	0.0895 (14)	0.0454 (8)	0.0572 (10)	−0.0206 (8)	0.0477 (10)	−0.0197 (7)
O5	0.1024 (15)	0.0491 (8)	0.0503 (10)	−0.0017 (8)	0.0441 (10)	0.0014 (7)
O6	0.0865 (13)	0.0525 (9)	0.0414 (8)	−0.0089 (8)	0.0358 (9)	−0.0143 (7)
O7	0.0853 (13)	0.0634 (9)	0.0410 (8)	−0.0037 (8)	0.0366 (9)	−0.0027 (7)

### Geometric parameters (Å, º)

**Table:**

C1—O3	1.346 (2)	C10—O6	1.357 (2)
C1—C2	1.380 (3)	C10—C11	1.424 (3)
C1—C6	1.396 (3)	C11—C12	1.421 (3)
C2—C3	1.372 (3)	C11—C13	1.445 (3)
C2—H2	1.02 (2)	C12—O4	1.353 (2)
C3—C4	1.380 (3)	C13—O7	1.247 (2)
C3—H3	0.91 (2)	C13—C14	1.487 (3)
C4—C5	1.381 (3)	C14—H14A	0.9600
C4—N1	1.447 (3)	C14—H14B	0.9600
C5—C6	1.375 (3)	C14—H14C	0.9600
C5—H5	0.91 (2)	N1—O1	1.222 (3)
C6—N2	1.405 (2)	N1—O2	1.222 (3)
C7—C12	1.374 (3)	N2—H2A	0.93 (3)
C7—C8	1.383 (3)	N2—H2B	0.93 (3)
C7—H7	0.98 (2)	O3—H3A	0.84 (3)
C8—O5	1.348 (2)	O4—H4	0.86 (3)
C8—C9	1.383 (3)	O5—H5A	0.88 (3)
C9—C10	1.371 (3)	O6—H6	0.92 (3)
C9—H9	0.95 (2)		
			
O3—C1—C2	123.81 (19)	O6—C10—C11	119.96 (18)
O3—C1—C6	115.21 (17)	C9—C10—C11	122.61 (18)
C2—C1—C6	120.98 (19)	C12—C11—C10	115.75 (17)
C3—C2—C1	120.3 (2)	C12—C11—C13	124.43 (17)
C3—C2—H2	115.1 (11)	C10—C11—C13	119.73 (17)
C1—C2—H2	124.5 (11)	O4—C12—C7	120.34 (17)
C2—C3—C4	118.2 (2)	O4—C12—C11	118.28 (17)
C2—C3—H3	121.8 (13)	C7—C12—C11	121.37 (17)
C4—C3—H3	120.0 (14)	O7—C13—C11	119.92 (17)
C3—C4—C5	122.63 (19)	O7—C13—C14	116.4 (2)
C3—C4—N1	118.4 (2)	C11—C13—C14	123.62 (19)
C5—C4—N1	118.9 (2)	C13—C14—H14A	109.5
C6—C5—C4	118.97 (19)	C13—C14—H14B	109.5
C6—C5—H5	119.0 (12)	H14A—C14—H14B	109.5
C4—C5—H5	122.0 (12)	C13—C14—H14C	109.5
C5—C6—C1	118.94 (17)	H14A—C14—H14C	109.5
C5—C6—N2	121.59 (19)	H14B—C14—H14C	109.5
C1—C6—N2	119.41 (18)	O1—N1—O2	121.9 (2)
C12—C7—C8	120.37 (18)	O1—N1—C4	119.5 (2)
C12—C7—H7	119.3 (11)	O2—N1—C4	118.7 (2)
C8—C7—H7	120.3 (11)	C6—N2—H2A	110.2 (14)
O5—C8—C9	117.50 (17)	C6—N2—H2B	112.7 (16)
O5—C8—C7	121.84 (18)	H2A—N2—H2B	112 (2)
C9—C8—C7	120.66 (19)	C1—O3—H3A	107 (2)
C10—C9—C8	119.21 (18)	C12—O4—H4	108.3 (16)
C10—C9—H9	119.9 (14)	C8—O5—H5A	112.0 (19)
C8—C9—H9	120.9 (14)	C10—O6—H6	107.3 (15)
O6—C10—C9	117.41 (17)		
			
O3—C1—C2—C3	−179.5 (2)	O6—C10—C11—C12	179.20 (18)
C6—C1—C2—C3	0.9 (4)	C9—C10—C11—C12	1.0 (3)
C1—C2—C3—C4	−1.2 (4)	O6—C10—C11—C13	2.4 (3)
C2—C3—C4—C5	0.9 (3)	C9—C10—C11—C13	−175.8 (2)
C2—C3—C4—N1	178.3 (2)	C8—C7—C12—O4	−177.8 (2)
C3—C4—C5—C6	−0.2 (3)	C8—C7—C12—C11	1.0 (3)
N1—C4—C5—C6	−177.64 (19)	C10—C11—C12—O4	177.22 (19)
C4—C5—C6—C1	−0.2 (3)	C13—C11—C12—O4	−6.2 (3)
C4—C5—C6—N2	177.0 (2)	C10—C11—C12—C7	−1.6 (3)
O3—C1—C6—C5	−179.80 (19)	C13—C11—C12—C7	175.0 (2)
C2—C1—C6—C5	−0.1 (3)	C12—C11—C13—O7	178.3 (2)
O3—C1—C6—N2	3.0 (3)	C10—C11—C13—O7	−5.2 (3)
C2—C1—C6—N2	−177.3 (2)	C12—C11—C13—C14	−2.9 (4)
C12—C7—C8—O5	−179.1 (2)	C10—C11—C13—C14	173.5 (2)
C12—C7—C8—C9	0.3 (3)	C3—C4—N1—O1	−1.4 (3)
O5—C8—C9—C10	178.5 (2)	C5—C4—N1—O1	176.1 (2)
C7—C8—C9—C10	−0.8 (3)	C3—C4—N1—O2	−180.0 (2)
C8—C9—C10—O6	−178.1 (2)	C5—C4—N1—O2	−2.5 (3)
C8—C9—C10—C11	0.2 (3)		

### Hydrogen-bond geometry (Å, º)

Cg1 and Cg2 are the centroids of the C7–C12 and C1–C6 rings, respectively.

**Table:**

*D*—H···*A*	*D*—H	H···*A*	*D*···*A*	*D*—H···*A*
N2—H2*A*···O6^i^	0.93 (3)	2.28 (3)	3.067 (2)	141.5 (19)
N2—H2*B*···O6^ii^	0.93 (3)	2.36 (3)	3.241 (3)	157 (2)
O3—H3*A*···N1^iii^	0.84 (3)	2.59 (3)	3.358 (3)	153 (3)
O3—H3*A*···O1^iii^	0.84 (3)	2.39 (3)	2.953 (3)	125 (3)
O3—H3*A*···O2^iii^	0.84 (3)	2.13 (3)	2.975 (3)	178 (3)
O4—H4···N2	0.86 (3)	1.94 (3)	2.784 (2)	166 (2)
O5—H5*A*···O7^i^	0.88 (3)	1.87 (3)	2.748 (2)	175 (3)
O6—H6···O7	0.92 (3)	1.64 (3)	2.478 (2)	150 (2)
N1—O2···*Cg*2^iv^	1.22 (1)	3.82 (1)	3.599 (3)	70 (1)
C13—O7···*Cg*1^v^	1.25 (1)	3.52 (1)	3.722 (3)	89 (1)

Symmetry codes: (i) *x*, −*y*, *z*+1/2; (ii) −*x*+1, −*y*, −*z*+1; (iii) *x*−1, −*y*+1, *z*−1/2; (iv) −*x*, *y*, −*z*+1/2; (v) −*x*+2, −*y*, −*z*+1.
